# Electroacupuncture for girdle sensation following spinal cord injury as a false localizing sign: a case series

**DOI:** 10.3389/fpain.2026.1794414

**Published:** 2026-05-22

**Authors:** Yuan Gao, Xinyi Su, Yang Yang, Qiang Liu, Shan Jing, Minke Liu

**Affiliations:** 1 Department of Acupuncture and Moxibustion, Zhejiang Rehabilitation Medical Center, Hangzhou, Zhejiang, China; 2 Department of Rehabilitation, West China Hospital of Sichuan University, Chengdu, Sichuan, China; 3 The Third Clinical Medical College, Zhejiang Chinese Medical University, Hangzhou, Zhejiang, China; 4 Department of Cardiopulmonary Rehabilitation, Zhejiang Rehabilitation Medical Center, Hangzhou, Zhejiang, China; 5 Department of Neurorehabilitation, Zhejiang Rehabilitation Medical Center, Hangzhou, Zhejiang, China; 6 Neurointerventional Department, The Affiliated Hospital of Gansu University of Chinese Medicine, Lanzhou, Gansu, China; 7 Encephalopathy Center, Affiliated Hospital of Gansu University of Traditional Chinese Medicine, Lanzhou, Gansu, China

**Keywords:** below-level neuropathic pain, electrocupuncture, false localizing sign, girdle sensation, spinal cord injury

## Abstract

**Background:**

Girdle sensation is a distressing false localizing sign that may occur following cervical spinal cord injury (SCI), often presenting as circumferential trunk constriction with burning pain and sleep disturbance. Effective treatments for this condition remain limited.

**Case summary:**

This case series describes two male patients (aged 52 and 45 years) who presented with girdle sensation following spinal cord injury. As symptoms were refractory to conventional pharmacological management with antispasmodics and analgesics, both patients underwent electroacupuncture (EA) at bilateral LU7 (Lieque), SI3 (Houxi), LI10 (Shousanli), and LI11 (Quchi). Symptom intensity and associated sleep disturbances were quantified utilizing the Visual Analogue Scale (VAS) and the Sleep Dysfunction Rating Scale (SDRS).

**Outcomes:**

Both patients reported immediate relief from the girdle sensation following the initial electroacupuncture (EA) session. Over the two-week treatment course, VAS and SDRS scores decreased progressively, reflecting sustained pain reduction and enhanced sleep. No adverse events were observed.

**Conclusion:**

This case series suggests that electroacupuncture is a promising non-pharmacological therapy for managing post-SCI girdle sensation. However, rigorous randomized controlled trials are warranted to validate its clinical efficacy and elucidate the underlying neuromodulatory mechanisms.

## 1 Introduction

Chronic neuropathic pain and paresthesia following spinal cord injury (SCI) are major sources of morbidity, affecting up to 64.9% of patients (1). Girdle sensation, a common clinical manifestation, presents as a circumferential, girdle-like tightness around the trunk and is frequently accompanied by burning neuropathic pain. In severe cases, it can significantly compromise respiration, sleep architecture, and functional rehabilitation (2). Although its exact incidence is not well-established, our institutional data indicate that this symptom affects approximately 50% of patients with cervical SCI. Classified as a false localizing sign, girdle sensation reflects a clinico-anatomical discordance, where symptoms manifest at segmental levels distant from the primary lesion or the anticipated neurological deficit (3). Beyond girdle sensation, cervical myelopathy may present with other false localizing signs, including complex regional pain syndrome (4), lower extremity sensory deficits (5), and radicular leg pain mimicking sciatica (3).

The underlying pathophysiology of girdle sensation remains incompletely understood, though it may involve multilevel spinal cord dysfunction. A recognized contributing factor is the compression of the midline ventral cervical cord (2), which disrupts ascending and descending white matter tracts, ultimately driving central sensory hypersensitivity (6). Currently, there are no definitively proven therapies for this condition, and high-quality evidence supporting acupuncture for post-SCI neuropathic pain remains scarce (7). Given this clinical challenge, we present our initial experience utilizing acupuncture for refractory girdle sensation, demonstrating substantial symptomatic relief.

## 2 Case presentation

### 2.1 Case 1

#### 2.1.1 Patient information and history

**Figure 1:**
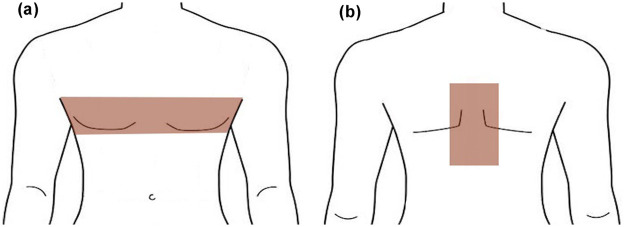
**(a)** schematic diagram of girdle sensation on the chest in case 1 and 2; **(b)** schematic diagram of girdle sensation on the back in case 2.

In October 2024, a 52-year-old male was admitted for inpatient rehabilitation three months after sustaining a traumatic C3-C4 cervical SCI. MRI revealed spinal edema and myelomalacia. His surgical history included decompression, tracheostomy, and subsequent decannulation. Prior to acupuncture, he reported a 6-cm-wide girdle-like constriction across his chest, accompanied by burning neuropathic pain that severely compromised his respiration and nocturnal sleep (Figure 1A). Physical examination demonstrated tetraparesis, sensory loss below the nipple line, and a positive bulbocavernosus reflex, consistent with incomplete SCI. To manage the refractory girdle-like sensation, he had been prescribed pregabalin (150 mg, twice daily) and baclofen (5 mg, three times daily), alongside neuromuscular electrical stimulation (NMES). However, these interventions provided limited relief, prompting his referral for acupuncture.

#### 2.1.2 Therapeutic intervention

**Figure 2:**
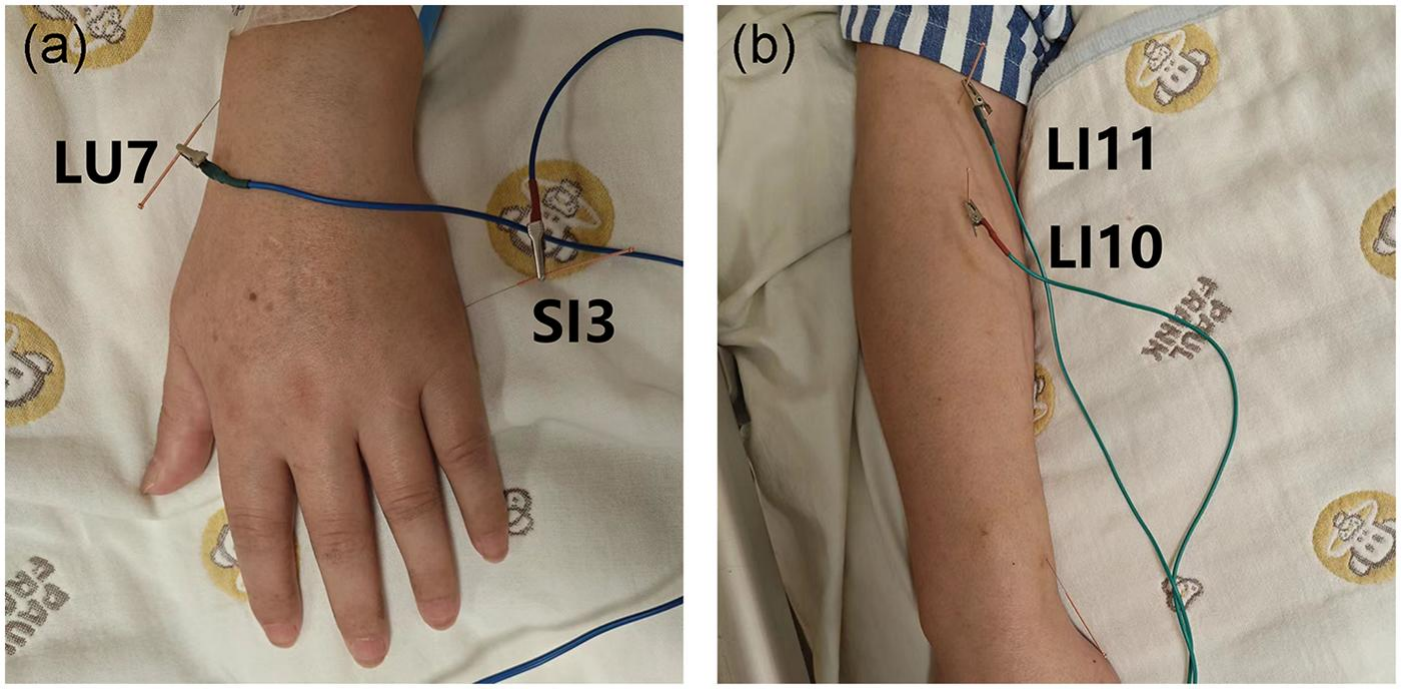
**(a)** acupoints of LU 7 and SI 3 used in case 1: **(b)** acupoints of LI 10 and LI 11 used in case 1.

Following clinical assessment, bilateral acupoints LU7 (Lieque), SI3 (Houxi), LI10 (Shousanli), and LI11 (Quchi) were selected. After local skin disinfection, sterile, single-use 0.25 mm × 40 mm stainless steel needles (Huatuo; Suzhou Medical Supplies Factory Co., Ltd., Suzhou, China) were inserted. Needle depths were 15 mm (transversely) at LU7, and 30 mm (perpendicularly) at SI3, LI10, and LI11. Continuous-wave electroacupuncture (EA) at 10 Hz was subsequently applied to the paired ipsilateral points (LU7-SI3 and LI10-LI11) for 30 min (Figure 2).

#### 2.1.3 Outcomes and follow-up

**Figure 3:**
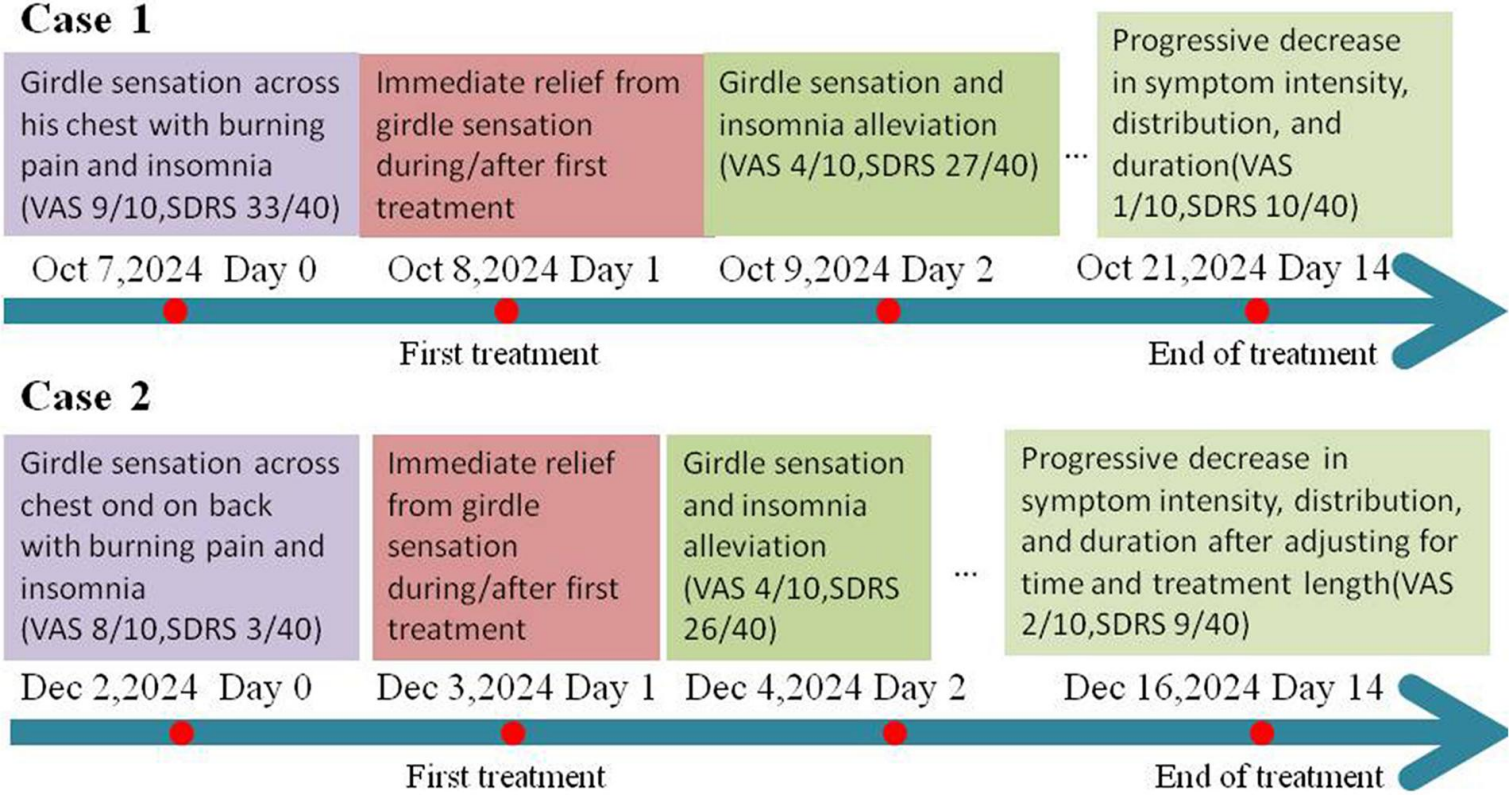
Timeline of brief outcomes during treatment.

**Figure 4:**
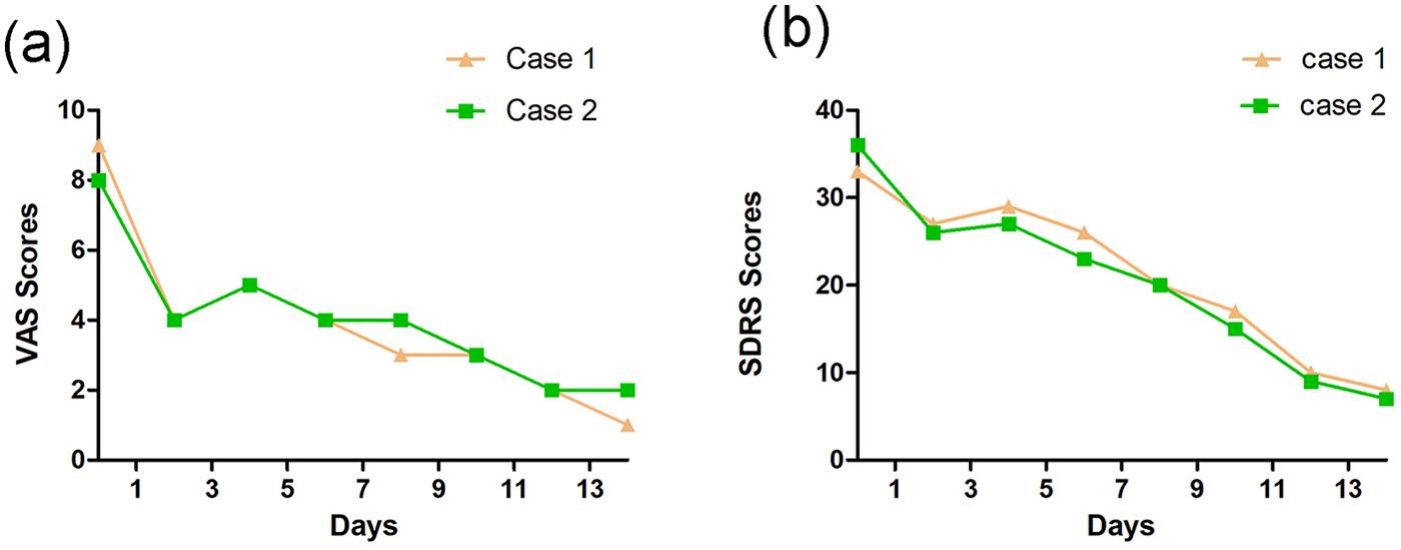
(**a**) VAS scores from each follow-up examination of two cases; (**b**) SDRS scores from each follow-up examination.

Within 10 min of treatment initiation, the patient reported an immediate reduction in the girdle-like constriction and burning pain, concomitant with increased respiratory amplitude. Symptom intensity was quantified using the Visual Analogue Scale (VAS) (8). Given the nocturnal exacerbation of his symptoms and resulting sleep disruption, the Sleep Dysfunction Rating Scale (SDRS) was employed to evaluate secondary insomnia (9). Baseline VAS and SDRS scores were 9/10 and 33/40, respectively. Following the initial EA session, the VAS score decreased immediately to 4/10, and the SDRS score fell to 27/40 the next morning, with the patient noting his first restful sleep since the injury. Throughout the subsequent two-week treatment course, the intensity, distribution, and duration of the neuropathic symptoms progressively diminished. By the end of treatment, VAS and SDRS scores had declined to 1 and 10, respectively (Figures 3, 4). The patient reported high satisfaction with the therapeutic outcome, and no adverse events were observed.

### 2.2 Case 2

#### 2.2.1 Patient information and history

In December 2024, a 45-year-old male was admitted for inpatient rehabilitation two months after sustaining a traumatic cervical SCI due to a fall from a height. MRI revealed C6–C7 vertebral fractures accompanied by spinal cord edema. His prior medical management included surgical decompression, tracheostomy, and subsequent decannulation. Prior to the acupuncture intervention, he reported a transverse, rope-like constriction across his chest associated with burning neuropathic pain. Unlike Case 1, he also experienced a longitudinal girdle-like tightness along his dorsum extending from C7 to T11, which further compromised his respiratory mechanics and sleep quality (Figures 1A, B). Physical examination demonstrated tetraparesis, sensory loss below the nipple line, and a positive bulbocavernosus reflex, consistent with incomplete SCI. The patient had undergone the same conventional pharmacological and rehabilitation regimen as Case 1, which yielded limited therapeutic benefit. Consequently, he was referred for acupuncture evaluation to manage these refractory neuropathic symptoms.

#### 2.2.2 Therapeutic intervention

The electroacupuncture (EA) protocol was identical to that administered in Case 1. However, given the chronicity and broader distribution of his girdle-like sensation, the session duration was extended to 60 min.

#### 2.2.3 Outcomes and follow-up

Consistent with Case 1, the patient experienced symptomatic relief in both the thoracic and dorsal girdle sensations following the initial EA session. A progressive decline in symptom intensity, spatial distribution, and duration was documented throughout the treatment course. By the conclusion of the therapy, substantial reductions were observed in both VAS (from a baseline of 8 to 2) and SDRS (from 36 to 9) scores (Figures 3, 4). Notably, the patient's nocturnal sleep remained severely disrupted when EA was administered in the morning. To address this, the daily treatment schedule was adjusted to 16:00. Following this chronotherapeutic modification, he reported markedly restored sleep quality and high satisfaction with the clinical outcome. The treatment was well tolerated, with no adverse events observed.

## 3 Discussion

In two patients with SCI whose chief complaint was refractory girdle sensation (a false localizing sign), conventional pharmacotherapy with antispasmodics and analgesics proved ineffective. Following the initial electroacupuncture (EA) session, both patients reported immediate symptomatic relief. A two-week course of EA substantially attenuated or completely resolved the thoracic constriction. This relief not only improved sleep quality but also facilitated daytime rehabilitation participation by alleviating the associated respiratory restrictions. These cases highlight that EA may offer a highly beneficial therapeutic option for SCI-induced girdle sensation.

Collier first described the false localizing sign in 1904 (10). Unlike conditions such as diabetic thoracic radiculopathy (11), polyradiculoneuropathy (12), neurotuberculosis (13), or neurosarcoidosis (14)—which typically affect the corresponding segmental levels—sensory deficits following cervical SCI often bypass the cervical region. Instead, they manifest at remote thoracic or lower levels, including the lumbar region, perineum, and lower extremities (15). This false localization of the sensory level may clinically obscure lesions at higher anatomical levels (16). In our cervical SCI cases, these false localizing signs manifested as girdle-like sensations across the anterior thorax and dorsum, consistent with previous literature (2). The anterior chest sensation was transversely distributed across 3–4 dermatomes from T3 to T11, while the dorsal sensation followed a longitudinal midline distribution.

The pathogenesis of the false localizing sign in cervical SCI, primarily characterized by girdle sensation, remains incompletely elucidated but may involve vascular factors (17). Ochiai et al. (2) postulated that this phenomenon could result from the compression of the midline ventral cervical cord or the anterior spinal artery. A recent case report (6) also indicated that girdle sensation in the middle trunk is associated with ventral cord compression, which causes spinothalamic tract dysfunction and subsequent sensory hypersensitivity. Furthermore, girdle sensation closely resembles below-level neuropathic pain—manifesting as pressure, constriction, and burning pain (18)—which typically occurs at least three segments below the level of neurological injury (19). Based on these observations, we hypothesize that girdle sensation may represent a specific trunk manifestation of below-level neuropathic pain. Most neuropathic pain below the SCI level occurs in the setting of incomplete SCI, where preserved axons in the white matter continue to conduct ascending and descending nociceptive signals (20). Compression of the midline ventral cervical cord likely disrupts these tracts, ultimately driving the hyperexcitability of lower-level spinal neurons (21).

Although literature specifically addressing acupuncture for post-SCI girdle sensation or below-level neuropathic pain remains scarce, existing evidence demonstrates promising clinical utility. For instance, in analogous neuropathic conditions such as phantom limb pain, both contralateral and ipsilateral acupuncture have been shown to effectively mitigate pain, likely via the modulation of cortico-spinal integration (22–24). Furthermore, a retrospective study by Rapson et al. (25) demonstrated that 1 Hz electroacupuncture (EA) applied at scalp acupoints significantly relieved bilateral symmetrical burning pain in patients with SCI. Similarly, Estores et al. (26) reported that auricular acupuncture effectively alleviated below-level neuropathic pain in the lower extremities. Building upon these findings, our observations suggest that EA offers distinct clinical advantages for managing girdle sensation, notably its rapid onset of action, favorable safety profile, ease of administration, and the critical practical benefit of not requiring patient repositioning. Given that girdle sensation frequently exacerbates at night, adjusting the timing or duration of treatment is critical for sustaining therapeutic effects and mitigating nocturnal symptoms (27). This chronotherapeutic approach aligns with our previous findings and may be intrinsically linked to the dose-response relationship of acupuncture (28).

Both presented cases involved incomplete SCI, characterized by preserved partial sensory function at the level of the lesion. We postulate that the therapeutic efficacy of EA for trunk girdle sensation is fundamentally rooted in the anatomical congruence between the selected acupoints and the sensory dermatomes of the cervical nerve segments. Specifically, Lieque (LU7) is located near the C6 dermatome on the radial aspect of the wrist; Houxi (SI3) corresponds to the C8 dermatome on the ulnar aspect of the proximal metacarpophalangeal joint; and Quchi (LI11) and Shousanli (LI10) map to the C5 dermatome on the radial aspect of the forearm. Physiologically, LU7 stimulation has been shown to enhance expiratory flow (29). Concurrent stimulation of SI3, LI10, and LI11 mitigates vasospastic contractions and muscle spasticity, reduces vascular resistance, and exerts positive neuromodulatory effects on the cervical spinal cord (30).

Consequently, we propose a dual-mechanism hypothesis for the therapeutic effects of EA at these specific acupoints. Neurologically, EA modulates aberrant nociceptive signaling within the ascending and descending sensory tracts of the spinal cord, thereby alleviating the painful and constrictive elements of the girdle sensation. Peripherally, at the effector organ level, it alleviates local muscle spasticity and optimizes respiratory mechanics, culminating in enhanced overall pulmonary function.

Beyond acupoint selection, the frequency of electroacupuncture (EA) and the total duration of the treatment course are critical determinants of clinical efficacy. Post-SCI girdle sensation constitutes a severe form of chronic neuropathic pain and allodynia, conditions closely associated with the dysregulation of central and peripheral neurotransmitter systems, including endogenous opioids. Opioid receptor agonists mediate analgesia by activating inwardly-rectifying potassium channels to induce membrane hyperpolarization. This hyperpolarization subsequently decreases neuronal excitability and inhibits presynaptic neurotransmitter release via the suppression of voltage-gated calcium channels (31). Preclinical evidence demonstrates that low-frequency (10 Hz) EA selectively activates *μ*- and *δ*-opioid receptors to produce potent analgesia (32). Notably, compared to high-frequency (100 Hz) stimulation, 10 Hz EA provides longer-lasting relief for inflammatory pain (33) and effectively attenuates nerve injury-induced hyperalgesia, a condition where 100 Hz EA demonstrates limited efficacy (34).

Despite these mechanistic insights, high-quality clinical evidence regarding optimal EA parameters for post-SCI neuropathic pain remains scarce (35). One longitudinal study reported improvements in pain intensity and associated sequelae in 46% of patients with SCI following 15 acupuncture sessions over a 7.5-week period (36). Aligning with this, our observations suggest that a condensed course of 7 EA sessions can provide significant symptomatic relief for girdle sensation, particularly when the treatment schedule is individualistically adapted—such as shifting the session timing to address specific nocturnal sleep disruptions.

While the clinical outcomes and proposed mechanisms of EA for post-SCI girdle sensation are highly encouraging, we acknowledge that this research remains in an exploratory phase. The primary inherent limitation of this report is its nature as a two-case series. Translating these preliminary observations into standardized evidence-based practice will necessitate adequately powered, sham-controlled randomized trials to definitively validate clinical efficacy and further elucidate the underlying neurobiological mechanisms.

## 4 Conclusion

In summary, this case series suggests that electroacupuncture represents a promising, non-pharmacological therapeutic option for the management of SCI-induced girdle sensation. Future research should further elucidate the precise neurobiological mechanisms underlying the effects of electroacupuncture on this false localizing sign, thereby facilitating the development of standardized, evidence-based treatment protocols.

## Data Availability

The raw data supporting the conclusions of this article will be made available by the authors, without undue reservation.
